# The pathogenesis of prevalent aerobic bacteria in aerobic vaginitis and adverse pregnancy outcomes: a narrative review

**DOI:** 10.1186/s12978-021-01292-8

**Published:** 2022-01-28

**Authors:** Xiaotong Ma, Ming Wu, Chen Wang, Huiyang Li, Aiping Fan, Yingmei Wang, Cha Han, Fengxia Xue

**Affiliations:** 1 Department of Gynecology and Obstetrics, Tianjin Medical University General Hospital, No. 154 Anshan Road, Heping District, Tianjin, 300052 China; 2 Tianjin Key Laboratory of Female Reproductive Health and Eugenics, Tianjin, China

**Keywords:** Vaginitis, Aerobic bacteria, Pregnancy outcome, Host–pathogen interactions, Therapeutics

## Abstract

**Background:**

Aerobic vaginitis is a common cause of vaginal discharge in reproductive-age women, increasing the risk of negative pregnancy outcomes such as premature delivery, abortion, premature rupture of membranes and stillbirth. However, the aetiology and pathogenesis of aerobic vaginitis causing negative pregnancy outcomes are still unclear, and there is no unified and standardized treatment method for aerobic vaginitis in the pregnancy period.

**Methods:**

We conducted a literature search of published studies in the English language focusing on aerobic vaginitis and its association with adverse pregnancy outcomes utilizing PubMed and Web of Science from January 1973 through June 2021. The common pathogenic bacteria of aerobic vaginitis during pregnancy, such as group B *Streptococcus*, *Escherichia coli*, *Staphylococcus aureus*, *Enterococcus faecalis* and *Klebsiella pneumoniae*, as well as the related adverse pregnancy outcomes and existing treatments were reviewed.

**Results:**

A total of 4534 articles were identified, and 97 studies that had inclusion criteria were subjected to careful review. The pathogenic bacteria of aerobic vaginitis can produce different toxins or affect the local immunity of patients and then lead to the occurrence of infection. Fresh wet mount microscopy is the preferred diagnostic method for aerobic vaginitis. Clindamycin is a common antibiotic used for aerobic vaginitis in pregnant women. The use of products combining probiotics has achieved excellent treatment success.

**Conclusions:**

Future research in this field can provide insights regarding the mechanism of aerobic vaginitis-induced adverse pregnancy outcomes in humans and ways to prevent their occurrence.

## Plain Language Summary

Aerobic vaginitis is an infection of the vagina that increases the risk of negative pregnancy outcomes. The aetiology and pathogenesis of aerobic vaginitis causing negative pregnancy outcomes are still unclear. This paper reviews the common pathogenic bacteria of aerobic vaginitis during pregnancy, and the related adverse pregnancy outcomes. We also review the existing treatment. Currently, it is believed that the microflora in aerobic vaginitis is composed of commensal aerobic microorganisms of intestinal origin, and the most frequently encountered bacteria are group B *Streptococcus*, *Escherichia coli*, *Staphylococcus aureus*, *Enterococcus faecalis* and *Klebsiella pneumoniae*. The pathogenic bacteria of aerobic vaginitis can produce different toxins or affect the local immunity of patients and then lead to the occurrence of infection. Fresh wet mount microscopy is the preferred diagnostic method for aerobic vaginitis. Clindamycin is a common antibiotic used for aerobic vaginitis in pregnant women. The use of products combining probiotics has achieved excellent treatment success. This study provides a reference for future research and early diagnosis and treatment during pregnancy. Future research in this field can provide insights regarding the mechanisms of aerobic vaginitis-induced adverse pregnancy outcomes in humans and ways to prevent their occurrence.

## Background

Aerobic vaginitis (AV) is a vaginal infectious entity caused by excessive commensal aerobic microorganisms of intestinal origin and is distinctly different from bacterial vaginosis, which is usually considered an endogenous infection [1]. It is one of the most common reproductive tract infections in women during the childbearing period. AV was first identified by the Belgian scholar Donders and colleagues in 2002 [2], and the principal diagnosis for identification of AV is currently observation of a wet film of vaginal secretion under a light microscope, combined with clinical manifestations [3]. The prevalence of AV is 7.9–23.7%, and the prevalence of AV during pregnancy is 4.1–8.3% [4–7]. Relevant studies were extracted from several research domains confirming that AV can cause adverse pregnancy outcomes such as premature delivery, abortion, premature rupture of membranes (PROM) and stillbirth, but the aetiology and pathogenesis of adverse pregnancy outcomes are still unclear [4]. This paper reviews the pathogenic bacteria and pathogenesis of common AV pathogens in pregnancy that may be related to adverse pregnancy outcomes to guide the clinical diagnosis and treatment of AV in pregnancy, reduce risk factors for adverse pregnancy outcomes, and protect the health of pregnant women and foetuses.

The pathogenic microbiota in the vagina of AV patients is very complex. A recent Indian study found that of the patients who presented with abnormal discharge, 15.0% were diagnosed with AV by culture, including infections with *Escherichia coli* (*E. coli*, 7.5%), *Staphylococcus aureus* (*S. aureus*, 4.5%), *Klebsiella* (2.0%), and *Enterococcus* species (1.0%) [8]. Another Indian study focused only on the isolation of aerobic bacteria in vaginal colonization in low-risk asymptomatic pregnant women with intact membranes who presented to a tertiary-level hospital for delivery; 52.6% of women had vaginal colonization with pathogenic aerobic bacteria, 13.2% of women yielded coagulase-negative *S. aureus*, and 8.9% of women had *E. coli* [9]. A Poland retrospective laboratory-based multi-centre study included patients with suspected vaginosis and clinical signs of infections and collected vaginal swabs. Ultimately, *Enterococcus faecalis* (*E. faecalis*, 29.2%) was the species most often isolated, followed by *E. faecalis* (29.2%), *E. coli* (26.3%) and *Streptococcus agalactiae* (*S. agalactiae,* 13.1%) [10]. Chen Wang et al. [11] tested the vaginal microbiota of nonpregnant AV patients by using 16S rRNA gene sequencing, a next-generation sequencing technique, and finally found that the relative abundance of some aerobes in the AV group subjects, including *S. agalactiae*, *Streptococcus anginosus* (*S. anginosus*), *Aerococcus christensenii*, *Streptococcus luteciae*, *Klebsiella pneumoniae* (*K. pneumoniae*), *E. coli*, *Streptococcus mitis*, *E. faecalis*, and *Pseudomonas putida*, increased markedly compared with that in healthy women.

## Methods

**Fig. 1:**
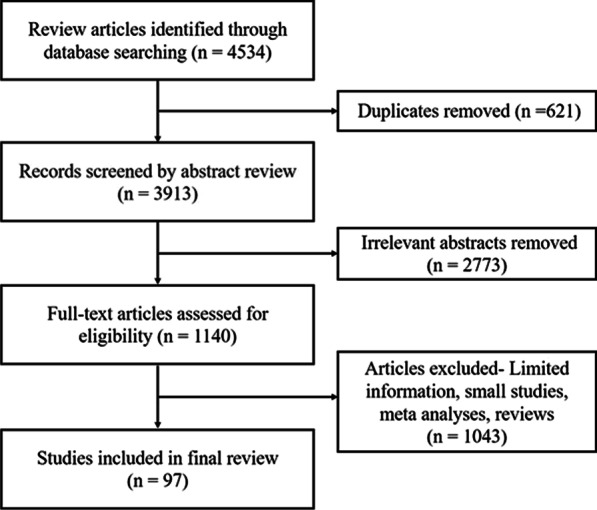
Flow diagram of the narrative review of the literature

A comprehensive search of PubMed and the Web of Science (until June 01, 2021) was conducted during this review. Search parameters, the MeSH indexing terms, included aerobic vaginitis, AV, *Streptococcus agalactiae*, *Escherichia coli*, *Staphylococcus aureus*, *Enterococcus faecalis*, *Klebsiella pneumoniae*, adverse pregnancy outcome, chorioamnionitis, PROM, preterm delivery, preterm pre-labour rupture of membranes, and stillbirth. All original and review studies in English based on the keywords searched in the database mentioned above, studies on humans and animal models, and studies published from 1973 to 2021 were included in our review. Non-English studies, conference abstracts, chapters of books, letters to the editor, and ex vivo studies were excluded. A total of 4534 articles from four databases were identified, and 97 studies that had inclusion criteria were subjected to careful review. Details of the search strategy are provided in Fig. 1.

## Results

### The relationship between common AV pathogens and adverse pregnancy outcomes

Currently, it is believed that the occurrence of AV is caused by abnormal vaginal microflora (AVF) or dysbiosis in the vaginal microenvironment [12, 13]. The microflora in AV is composed of commensal aerobic microorganisms of intestinal origin, and the most frequently encountered bacteria are *S. agalactiae* (group B *Streptococcus*, GBS), *E. coli*, *S. aureus*, *K. pneumoniae* and *E. faecalis* [3, 4, 11, 13–15]. The common aerobic microflora of AV found in different countries and regions are slightly different, and the influence of different pathogens on pregnancy outcome is different.

#### GBS and its adverse pregnancy outcomes

GBS, as a normal microbiota constituent, exists in the female genital tract and anal areas of healthy adults [16, 17]. The global colonization rate of GBS is 7.9%, and a study found that each region was slightly different; the highest colonization rate was 22.4% in Africa, followed by 19.7% in the Americas, 19% in Europe, and 11.1% in Southeast Asia [18]. However, research data in China are still lacking [19]. It has been reported that 7–25% of women between 35 and 37 weeks of gestation with AV are colonized with GBS, and intrauterine infection is related to ascending infection and the colonization ability of GBS in the lower reproductive tract [20–22]. Currently, GBS infection is the foremost cause of neonatal mortality and morbidity in the world [23–25]. GBS colonization in the vagina during pregnancy can cause chorioamnionitis, PROM, and postpartum endometritis by ascending infection, thereby increasing the occurrence of preterm delivery, preterm pre-labour rupture of membranes, and stillbirth, resulting in neonatal sepsis and meningitis [23, 26].

Studies have shown that GBS achieves ascending infection by inducing glycogen synthase kinase 3β(GSK3β) phosphorylation. In vitro and in mice, it has been found that GBS activates integrins and induces GSK3β phosphorylation after vaginal colonization; vaginal epithelial adherens junctions break down; β-catenin is released into the cytoplasm and interacts with dephosphorylated GSK3β, leading to β-catenin nuclear translocation; β-catenin can enter the nucleus and stimulate the expression of a variety of genes, including those that drive epithelial-to-mesenchymal transition and epithelial exfoliation; and epithelial exfoliation permits GBS dissemination through the loss of barrier function, leading to increased ascending infection, which increases the rates of adverse pregnancy outcomes and preterm birth [27]. GBS extracellular fibrinogen-binding proteins interact with host laminin and fibrinogen, leading to increased adherence to cervicovaginal epithelial cells and biofilm formation [28]. GBS can also generate haemolytic pigment, which promotes GBS penetration of the human placenta, and induce loss of barrier function in human amniotic epithelial cells, causing significant uterine contractions and preterm birth [29, 30]; the haemolytic pigment also stimulates the K^+^ outflow of red blood cells and macrophages, leading to cytolysis or pyroptosis and exacerbating preterm birth or foetal damage [31]. GBS hyaluronidase, known as HylB, promotes vaginal colonization, is secreted by GBS and specifically targets and degrades host hyaluronic acid into its disaccharide components, which are immunosuppressive, as they bind to Toll-like receptor (TLR)2/TLR4 receptors and block signalling [32, 33]. In addition, GBS can also generate extracellular membrane vesicles, which can lead to leucocyte infiltration and choriodecidual tissue inflammation in pregnant mice, resulting in premature delivery and foetal injury [15]. GBS can stimulate the NLRP3 inflammasome in a number of immune cells, such as dendritic cells, macrophages and neutrophils, which leads to the secretion of IL-1β and other inflammatory cytokines. GBS can also make use of its haemolysin to permeate the macrophage cell membrane, activate the NLRP3 inflammasome in macrophages, and then cause pyroptosis and foetal damage [31].

#### *Escherichia coli* and its adverse pregnancy outcomes

*Escherichia coli* linked to AV is extraintestinal *E. coli*. At present, *E. coli* infection is associated with abortion, PROM, premature delivery, stillbirth and other adverse pregnancy outcomes. *E. coli* inoculated into the vagina of pregnant mice has been found to ascend into the uterine cavity and induce premature delivery [34]. Moyo et al. [35] studied 104 pregnant women with stillbirth of unknown aetiology in Zimbabwe and 96 age- and parity-matched referents with normal full-term live foetuses; collected placental, amniotic fluid and foetal internal tissues; and found that *E. coli* (OR = 11.5, 95% CI 2.2–61), *Treponema pallidum* (OR = 8.3, 95% CI 2.8–25) and *Toxoplasma gondii* (OR = 3.9, 95% CI 1.9–7.8) were the main risk factors for stillbirth. Folgosa et al. [36] revealed a case-match study case-referent study carried out on 58 cases of pregnant women with late foetal death and 58 cases of pregnant women at term with a live foetus matched for age and parity in Maputo, Mozambique; collected samples from pregnant women and stillborn and liveborn foetuses for microbiological and histological assessment; and found that chorioamnionitis was associated with stillbirth, that the chorioamnionitis rate of pregnant women with stillbirth was higher than that of pregnant women with a live foetus (96% vs 67%), that *E. coli* was the species most frequently isolated in stillborn foetuses and was associated with chorioamnionitis in 28% of the stillborn foetuses as compared to 5% of the referents (OR = 6.9, 95% CI 1.4–65.4), and that vasculitis was found in 21% cases of stillborn foetuses, suggesting that the foetus was alive at the onset of infection. Another case-referent study was carried out on 58 cases of pregnant women with foetal death of unknown aetiology and 116 cases of pregnant women at term with live foetuses in Lithuania; collected samples from the maternal endocervix, amniotic fluid, placenta, external ear, and newborn blood; and histologically examined the placentas; and found that 36% of the stillbirth foetus had bacteraemia, that 21% of bacteraemic blood had *E. coli* as the most common strain, and that 50% of *E. coli* infectious cases associated with chorioamnionitis, prompting foetal bacteraemia, were significantly correlated with maternal placenta and membrane inflammation [37]. In southern Sweden, Tolockiene et al. [38] conducted a study with 117 cases of pregnant women with late foetal death and 126 cases of pregnant women at term with live foetuses; collected control newborn and stillbirth foetal placental and outer ear swabs; collected foetal lung, heart, blood, liver and brain from the stillborn foetuses; and found that extension of the inflammation to the decidua basalis was seven times more common among the stillbirth group than among the control group (OR = 7.2, 95% CI 2.8–21.9), that the risk of stillbirth was doubled when both inflammation and bacteria were present (OR = 2.3, 95% CI 0.92–5.8), and that *E. coli* infection in internal tissues of stillborn foetuses (such as the lungs, heart, blood, liver and brain) or in the placenta might be the main cause of death. Australian scholars McDonald et al. [39] conducted a 4-year retrospective review of 129 cases of spontaneous mid-gestation abortions; 85 (66%) cases had bacterial infection in the placentas and foetuses, and *E. coli* was isolated in a quarter (22 cases) of these cases. In Holdsworth Memorial Hospital, Mysore, South India, a hospital-based case–control study of 150 patients presenting with PROM and 150 controls matched for age, gestational age and parity was conducted, finding that *E. coli* in vaginal fluid was positively associated with PROM (OR = 7.5, 95% CI 2.8–20.0) and that vaginal infection caused by *E. coli* was an independent risk factor for PROM [40]. In Yale-New Haven Hospital, Pettker et al. [41] conducted a prospective study of 56 women who delivered within 48 h, and 23 patients assessed for foetal lung maturity in the third trimester served as controls. The results showed that 8% of patients with PROM had *E. coli* infection, which is the most common aerobic bacterial infection. Studies have shown that there is a significant correlation between the presence of *E. coli* in vaginal or cervical fluid and preterm delivery [42–44]. A retrospective study involving 36 studies and 3782 pregnant women with PROM found that the two primary microorganisms isolated from women with PROM and their infants were *S. aureus* and *E. coli* at 17.0% (643/1706) and 5.4% (204/1704), respectively [43]. Bacterial infection of the lower genital tract can stimulate the production of chemokines, cytokines, proteases and inflammatory mediators, thus initiating the contractile force of the myometrium [44].

The adverse pregnancy outcomes caused by *E. coli* are related to the strain type and virulence factors. Under normal circumstances, intestinal and vaginal symbiotic *E. coli* rarely cause disease. Some types of *E. coli*, however, acquire specific toxic properties, enhance adaptability, and then cause a wide range of diseases [45]. Pathogenicity-associated islands (PAIs) are a new concept in the study of bacterial pathogenesis; these cluster on mobile genetic elements and produce new pathogenic bacteria and evolutionary factors [46]. Virulence factors are successfully carried on PAIs, which then change *E. coli* into specific pathogen types that can cause diseases [47, 48]. The hydroperoxidases HP I and HP II produced by *E. coli* can hydrolyse hydrogen peroxide into water and oxygen, resisting the functions of hydrogen peroxide and protecting *E. coli* from damage, finally allowing *E. coli* to proliferate [49, 50]. At low concentrations of hydrogen peroxide, HP I is synthesized from the *katG* gene after activation by transcription factor OxyR binding. In the stable growth phase of bacteria, HP II is encoded by the *katE* gene after the RpoS protein (σ^s^ factor) encoded by *katF* (or *rpoS*) gene binds with RNA polymerase and then helps *E. coli* resist hydrogen peroxide [50, 51]. Adhesins can be produced by *E. coli*, mainly including type 1 fimbria, P fimbria, S fimbria and Dr fimbria. Type 1 fimbria is the key structure for *E. coli* strains to adhere to epithelial cells and is widely found in *E. coli* [52]. P fimbria is also widely found in *E. coli* and is considered to be the key virulence factor for *E. coli* to continue ascending infection [53]. Invasion of brain endothelium A is another virulence factor in *E. coli*, and studies have shown that it is responsible for invasion of brain microvascular epithelial cells and neonatal meningitis in humans [54]. It remains to be determined whether the type of *E. coli* pathogen associated with adverse pregnancy outcomes exists in the cervicovaginal or upper female genital tract. Watt et al. [45] isolated *E. coli* strains from various parts of pregnant women, such as the vaginas of asymptomatic pregnant women, amniotic fluid of pregnant women with chorioamnionitis and asymptomatic newborns, faeces of asymptomatic pregnant women, and blood or cerebrospinal fluid of newborns with neonatal sepsis; compared the various strains; and found that *E. coli* B2 strains isolated from the vagina, amniotic fluid, blood and cerebrospinal fluid were significantly correlated, whereas those isolated from faeces were not. Chong Fan et al. [55] developed a mouse model of vaginal infection induced by *E. coli* to mimic AV after pregnancy and found that vaginal infection with *E. coli* in pregnancy could cause adverse pregnancy outcomes. The mechanisms of the effects of vaginal infections induced by *E. coli* on pregnancy outcome revealed that macrophages located at the maternal-foetal interface might have a crucial effect and further affect pregnancy outcomes through the IL-4/JAK-1/STAT-6 signalling pathway. Compared with those in the vaginal tracts of mice in the control group, there were fewer macrophages of the M2 phenotype in the vaginal tracts of mice in the study group, and the levels of the corresponding chemokines, including CCL-17, CCL-22, and CCL-24, were reduced. The downstream gene expression of IL-4, JAK-1, STAT-6, and GATA-3 then all decreased significantly at different levels.

#### *Staphylococcus aureus* and its adverse pregnancy outcomes

Risk factors for vaginal colonization of *S. aureus* during pregnancy and the relationship between maternal vaginal colonization and infant infection are not well understood [56]. Pimentel et al. [57] carried out a study of community-related methicillin-resistant *S. aureus* (MRSA) infection causing PROM and neonatal sepsis eventually leading to chorioamnionitis and neonatal death. *S. aureus* infection accounts for over 90% of late-onset sepsis in neonates. Late-onset *S. aureus* sepsis is four times more frequent in very low birth-weight infants than in normal infants [58]. Doster et al. [59] conducted an experiment with *S. aureus* and gestational membrane samples cocultured for 24 h, and the results demonstrated that *S. aureus* penetrated the chorion but did not traverse the amnion. *S. aureus* can grow and form biofilms on the choriodecidual surface of gestational membranes and significantly increase IL-1β, IL-2, IL-6, GM-CSF, IFN-γ, and TNF-α levels.

Currently, there are a few studies on the mechanism of *S. aureus* and adverse pregnancy outcomes. Studies have shown that *S. aureus* can secrete Panton-Valentine leukocidin (PVL) exotoxin, which can form a polymer with a ring structure to insert into the cell membrane and form 2 nm apertures, selectively allowing divalent cations such as Ca^2+^ and Mg^2+^ to cross and then accelerating the necrosis and apoptosis of white blood cells [60]. Changes in Ca^2+^ concentration can simultaneously activate a variety of transcriptional regulators [61]. Increased Ca^2+^ concentrations can activate intracellular calmodulin, a Ca^2+^/calmodulin-dependent protein phosphatase, which activates IκB kinase and then inactivates IκB from NF‐κB, thereby increasing the amount of nuclear NF‐κB DNA-binding activity and participating in transcriptional regulation of various inflammatory factors [62]. Studies have reported that increased levels of IL-6 and IL‐8 can be induced by PVL, which are the major regulatory inflammatory factors of NF‐κB, and can induce granulocyte activation [63]. *S. aureus* can also produce clumping factor A, which is the main protein binding to fibrinogen and determines the binding capacity of *S. aureus* and fibrinogen [64]. Studies have shown that the activity of clumping factor A binding fibrinogen is crucial to the pathogenicity of *S. aureus* because clumping factor A can make *S. aureus* stick to the infected site and then cause disease by local infection. At the same time, after binding with fibrinogen, clumping factor A wrapped by fibrinogen can inhibit opsonins from approaching its surface to realize anti-phagocytosis activity [65].

#### *Enterococcus faecalis* and its adverse pregnancy outcomes

*E. faecalis* is a common cause of genitourinary infections, subacute bacterial endocarditis, abdominal abscesses, and wound infections [66, 67]. Kazimierak et al. [68] carried out a study of 244 pregnant women in the Department of Pathology of Pregnancy at Medical University in Lódź to collect cervical secretions and found that only 2% of pregnant women’s cervical secretion cultures were negative and that the most common bacterium isolated from cervical secretions was *E. faecalis*. Hufnagel et al. [66] performed a prospective epidemiological study of 274 infants admitted to a neonatal intensive care unit in the University Medical Center Freiburg in Germany and their mothers; collected specimens from the ear, pharynx, gastric content and first meconium of infants as well as rectovaginal swabs from pregnant women between weeks 35 and 37 of their pregnancies; and found that enterococcus colonization in infants was related to premature birth. Furthermore, in neonates, *E. faecalis* is associated with a 6% mortality rate in early-onset septicaemia, which increases to 15% in late-onset septicaemia [67], while infection with *E. faecalis* is implicated in 7% to 50% of fatal cases [69]. The effect of *E. faecalis* on neonatal necrotizing enterocolitis is still unclear. Seliga-Siwecka et al. [70] carried out a prospective cohort study of 396 neonates born at 22–32 weeks of gestation; collected placentas and umbilical cords for pathological evaluation and swabs of genital tract and amniotic fluid for bacterial culture; and found that the presence of *E. faecalis* in the amniotic fluid greatly increased the risk for placental tissue inflammation processes (OR = 10.7, 95% CI 1.27–89.9) and increased the risk for bronchopulmonary dysplasia and necrotizing enterocolitis. However, scholars have found that the colonization rate of *E. faecalis* in premature neonates is significantly lower than that in healthy neonates and that *E. faecalis* can reduce the secretion of the pro-inflammatory factor interleukin 8 (IL-8) by inhibiting the SAPK/JNK and P38 MAPK signalling pathways, thus reducing the occurrence of necrotizing enterocolitis in neonates [71, 72].

There are a few studies on the mechanism of *E. faecalis* and adverse pregnancy outcomes. Cytolysin can be produced by *E. faecalis*, which is encoded by genes carried on pheromone-responsive plasmids or chromosomes of *E. faecalis*, and a gene cluster containing six genes, including the structural genes *cyl*L_L_ and *cyl*L_S_, is required for post-translational modification enzyme *cyl*M, transporter and protease *cyl*B, activating protease *cyl*A and *cyl*I [73, 74]. Cytolysin can lyse some gram-positive organisms, erythrocytes, and other eukaryotic cells by making small pores appear on the cytoplasmic membrane, leading to rupture of the cytoplasmic membrane and small-molecule leakage, finally causing host cell damage and aggravating the degree of *E. faecalis* infection [75]. *E. faecalis* can also increase *tst* gene expression, leading to increased production of toxic shock syndrome toxin-1 (TSST-1) and thereby increasing the virulence of *S. aureus* [76].

#### *Klebsiella pneumoniae* and its adverse pregnancy outcomes

Currently, there are relatively few studies on the mechanism of *K. pneumoniae* infection and pregnancy outcomes. In 2005, Sheikh et al. [77] first reported a case of acute suppurative placentitis caused by *K. pneumoniae*, resulting in foetal demise at 18 weeks of gestation, and they speculated that severe infections that invade the uterine cavity are caused by *K. pneumoniae* colonization in the vagina. Omwandho et al. [78] and Torabi et al. [79] reported cases of early intrauterine foetal death caused by intrauterine *K. pneumoniae* infections. Carey et al. [12] studied the vaginal microbiota of pregnant women during pregnancy and delivery and found that an increase in *K. pneumoniae* levels in the vagina was an independent risk factor for preterm delivery. Seliga-Siwecka et al. [70] conducted a prospective cohort study of neonatal outcomes of premature delivery in women with abnormal genital tract colonization and chorionic amniotic inflammation and found that *K. pneumoniae* can increase the risk of chorioamnionitis, as well as the risk of respiratory distress syndrome and patent ductus arteriosus.

*K. pneumoniae* can produce a kind of acidic lipopolysaccharide named capsular polysaccharide (CPS). CPS can protect organisms from phagocytosis by macrophages, neutrophils, epithelial cells and dendritic cells and inhibit early inflammatory responses, mainly by inhibiting the expression of IL-8 in epithelial cells by inhibiting the TLR2/TLR4 signalling pathway and nucleotide-binding oligomerization domain containing 1 (NOD1). CPS can also affect the maturation of dendritic cells and reduce the production of pro-Th1 cytokines, such as IL-12 and tumour necrosis factor α (TNF-α), and then affect antigen presentation of immature dendritic cells and suppress T cell activation and natural killer cell migration, finally helping organisms escape the host immune system [80, 81].

### Diagnosis of AV

As Gilbert G G Donders et al. [2] described, fresh wet mount microscopy is the preferred diagnostic method, showing vaginal smears diminished or deficient in lactobacilli, with the presence of cocci or small coliform bacilli, parabasal epithelial cells, and toxic leukocytes. Lactobacillary grade (LBG), number of leukocytes, proportion of toxic leukocytes, background flora and proportion of parabasal epithelial cells (PBCs) are evaluated, and a score ranging from 0 to 2 is assigned to each of the five abovementioned characteristics, which are added together to calculate a composite score. AV is then diagnosed according to the composite score, as follows: a score of 0–2 means no AV; a score between 3 and 4 indicates light AV, a score between 5 and 6 represents moderate AV, and a score from 7 to 10 represents severe AV. Wang et al. [12] developed another test for AV diagnosis. AV was diagnosed based on five enzymatic indicators: (1) hydrogen peroxidase activity; (2) leukocyte esterase activity; (3) sialidase activity; (4) β-glucuronidase activity; and (5) coagulase activity. The reported sensitivity was 90%, but the study lacked analysis of specificity. Rumyantseva et al. [5] evaluated the use of quantitative polymerase chain reaction (qPCR) for the diagnosis of AV. Using mathematical formulas, including the concentration of *lactobacilli* and aerobic and anaerobic microorganisms as variables, they were able to accurately detect cases of AV. However, the authors recognized the limitations of qPCR for the diagnosis of AV because inflammation and immaturity of epithelial cells were not accounted for. A shortcoming of the study was its lack of sensitivity and specificity analysis. Although most women with AV have positive cultures for aerobic bacteria such as *Streptococcus agalactiae*, *S. aureus*, and *E. coli*, a positive vaginal culture does not indicate that the woman has AV and is not recommended for diagnosis [82]. However, culture with antimicrobial susceptibility testing may aid in treatment. Tempera et al. [83] diagnosed AV on the basis of the following parameters: (1) abnormal yellow vaginal discharge; (2) foul smell; (3) pH > 5; (4) abundant vaginal leukocytes on microscopic examination; and (5) LBGs of IIa, IIb and III. LBG of IIa, IIb and III were determined using Donders classification [84]. Grade II corresponds to a diminished lactobacillary flora mixed with other bacteria, and is subdivided into IIa slightly disturbed, fairly normal and IIb moderately disturbed and rather abnormal lactobacillary flora. Grade III is defined as an abnormal flora that consists of numerous other bacteria with absence of lactobacillary flora. This diagnostic method combines the clinical manifestations with microscopic examination. However, quantitative standards of clinical manifestations are lacking; thus, few clinical applications of the diagnostic criteria are available.

### Treatment of AV during pregnancy

AV is a notorious cause for pregnant women to have adverse pregnancy outcomes that can not only impair maternal and infant health but also lead to adverse pregnancy outcomes and affect the health of newborns [85]. Therefore, early detection, early diagnosis and early treatment are necessary for women with AV during pregnancy. However, studies on the treatment of AV during pregnancy are very limited, and only a few articles on the treatment of AV during pregnancy are available. AV is treated with antibiotics with intrinsic activity against aerobic bacteria, in addition to ensuring minimal interference with vaginal Lactobacillus species. For pregnant women, a labelling system described by the US Food and Drug Administration (FDA) Pregnancy and Lactation Labeling Rule (PLLR) containing a 5-tier set of alphabetic categories (ABCDX) is widely used to designate the safety of a drug for use during pregnancy [86]. Clindamycin is a broad-spectrum antibiotic that can cover gram-positive bacterial and anaerobic bacterial infections and can reduce the incidence of infection-related premature delivery [87–89]. As a category B drug, clindamycin is a common antibiotic used for AV infection in pregnant women [90]. Mumtaz et al*.* [91] conducted in vitro culture experiments and determined susceptibilities to various antibiotics of 1923 vaginal swabs from AV patients, finding that carbapenems and β-lactam-β-lactamase inhibitor combinations were the most effective antibiotics; moreover, as a category B drug, it could be considered for application to AV patients during pregnancy. Studies have shown that moxifloxacin, a fourth-generation quinolone, shows broad-spectrum antibacterial activity, including against gram-positive bacteria, gram-negative bacteria, anaerobic bacteria and atypical organisms such as mycoplasma, chlamydia and legionella, and shows a significant curative effect on nonpregnant AV patients [4, 92]. However, as a category C drug, moxifloxacin can decrease foetal body weight and slightly delay foetal skeletal development, so moxifloxacin should be used during pregnancy only if the potential benefit justifies the potential risk to the foetus. Kanamycin can cover gram-negative bacteria such as *E. coli*, *K. pneumoniae* and *S. aureus* infection, while local drug administration of kanamycin has good intestinal activity against gram-negative bacteria and natural inactivity against lactobacilli and has been confirmed to have therapeutic efficacy for nonpregnancy AV [83, 93]. However, kanamycin is a category D drug that can enter foetal tissues through the placental barrier and then cause irreversible foetal ototoxicity, so doctors must fully weigh the pros and cons before use. In recent years, the use of products combining probiotics has achieved excellent treatment success, and the function of probiotics in stabilizing vaginal flora has received wide attention. Women with abnormal vaginal flora can take probiotics orally or vaginally to improve the conditions of the vaginal flora [94–97].

### Future research recommendations

It is necessary to conduct epidemiological studies to trace the pathogens most commonly found in AV to better direct clinicians toward the optimal therapeutic choices. The review revealed that the association of AV with adverse pregnancy outcomes has not been as widely researched as bacterial vaginosis (BV) and needs further investigation. Since AV has implications for both pregnant women and unborn children, it needs to be identified and managed in an optimal manner, and it is necessary to develop products that are simple to apply, inexpensive and easy to handle. In the actual context of antibiotic resistance spread, it is fundamental to administer correct antibiotics, especially accounting for the local epidemiological spread. Furthermore, the frequent misdiagnosis of AV coupled with the emerging antimicrobial resistance associated with bacteria implicated in AV and neonatal nosocomial infections pose a problem for prophylaxis and treatment to reduce the risk of maternal and neonatal morbidity and mortality. AV is a mixed infection of vaginal aerobacteria, and the pathogenesis is still unclear. Further study of the pathogenesis of vaginal AV infection in pregnant women is expected to further halt the progression of the disease and ensure the outcomes of mother and child.

## Limitations

If documents were not indexed in the academic databases accessed for this review or searched through the Internet or websites, relevant articles that met the review criteria may have been omitted. Relevant articles published before the year 1980 or those that were not written in English were excluded and may have contained additional information about the aerobic vaginitis during pregnancy and related adverse pregnancy outcomes. Indeed, AV was not always reported or was not reported consistently. Studies on AV during pregnancy were very limited, most of which focused on the composition of vaginal flora in patients with AV and its impact on pregnancy outcomes. At the same time, there are relatively few in vitro or animal experiments on the pathogenesis of AV. Animal models of AV are not yet sound. The pathogens and induced infection are different, and the results can change. A single strain can account for only one of the pathogenic mechanisms. AV is mostly mixed infection of aerobic bacteria, and its pathogenic mechanism may be more complex. Recurrent infections are common in clinical practice, but it was not possible to further investigate them in this study. Therefore, a comparative study of recurrent infections, including frequency and timing of infection, diagnostic methods and other detailed questions, is necessary.

## Conclusions/Discussion

This review has explored the literature on the effects of AV on pregnancy outcomes and studies on the pathogenesis of common AV pathogens. The conducted literature review suggests that AV is an important factor leading to adverse pregnancy outcomes in pregnant women. Bacteria in AV, such as GBS, *E. coli*, *S. aureus*, *K. pneumoniae* and *E. faecalis*, cause a series of adverse pregnancy outcomes, including premature delivery, abortion, PROM and stillbirth. However, the mechanism by which AV leads to adverse pregnancy outcomes is unclear. A large number of in vitro and animal experiments have revealed possible pathogenic mechanisms of AV-associated bacteria, but they have not been confirmed in humans. In the future, we need to further study the mechanism of AV-induced adverse pregnancy outcomes in humans to find ways to prevent their occurrence. We also need to conduct research on early diagnosis and effective treatment for AV during pregnancy, to provide evidence-based medical advice, ultimately ensure the health of pregnant women and foetuses, and provide a new strategy to improve population health.

## Data Availability

Not applicable.

## Abbreviations

AV: Aerobic vaginitis
AVF: Abnormal vaginal microflora
PROM: Premature rupture of membranes
*E. coli*: *Escherichia coli*
*S. aureus*: *Staphylococcus aureus*
*E. faecalis*: *Enterococcus faecalis*
*S. agalactiae*: *Streptococcus agalactiae*
*S. anginosus*: *Streptococcus anginosus*
*K. pneumoniae*: *Klebsiella pneumoniae*
GBS: Group B *Streptococcus*
GSK3β: Glycogen synthase kinase 3β
TLR: Toll-like receptor
PAIs: Pathogenicity-associated islands
MRSA: Methicillin-resistant *S. aureus*
PVL: Panton-valentine leucocidin
IL-8: Interleukin 8
TSST-1: Toxic shock syndrome toxin-1
CPS: Capsular polysaccharide
NOD1: Nucleotide-binding oligomerization domain containing 1
TNF-α: Tumor necrosis factor α
LBG: Lactobacillary grade
PBCs: Parabasal epithelial cells
qPCR: Quantitative polymerase chain reaction
FDA: Food and Drug Administration
PLLR: Pregnancy and Lactation Labeling Rule
